# Neoadjuvant hyperthermic intraperitoneal chemotherapy: impact on chemotherapy response score and prognosis in high-grade serous ovarian carcinoma

**DOI:** 10.3389/fonc.2025.1639810

**Published:** 2025-09-09

**Authors:** Xiaojie Feng, Yuhua Wang, Han Li, Ye Zhang, Lei Li, Yanying Ma, Shanping Wang, Li Wang

**Affiliations:** ^1^ Department of Gynecologic Oncology, The Affiliated Cancer Hospital of Zhengzhou University & Henan Cancer Hospital, Zhengzhou, China; ^2^ Department of Pathology, The Affiliated Cancer Hospital of Zhengzhou University & Henan Cancer Hospital, Zhengzhou, China; ^3^ School of Biomedicine, Guangdong University of Technology, Guangzhou, China

**Keywords:** HGSOC, NHIPEC, NACT, CRS, prognosis

## Abstract

**Background:**

This study aimed to assess the therapeutic effects of neoadjuvant hyperthermic intraperitoneal chemotherapy (NHIPEC) combined with neoadjuvant chemotherapy (NACT) in patients with high-grade serous ovarian carcinoma.

**Methods:**

This study used a retrospective cohort design. A total of 120 patients with ovarian cancer who received NACT and underwent interval debulking surgery (IDS) at our hospital between 2016 and 2018 were enrolled in this study. Thereinto, 67 patients received the NHIPEC combined with the NACT regimen, and the remaining 53 patients received only NACT as the control. The degree of tumor burden reduction in patients treated with different regimens was evaluated using the chemotherapy response score (CRS), and patient survival data were analyzed.

**Results:**

We found that the combination with NHIPEC is independently and positively correlated with a CRS reaching grade 3 (P=0.002), indicating that the combined treatment regimen has a significant advantage in improving the tumor response rate. Moreover, NHIPEC is an independent favorable prognostic factor for overall survival (OS) (P=0.032) and progression-free survival (PFS) (P=0.029). Further analysis showed that the median OS and PFS of patients receiving the combined treatment regimen were extended to 40 and 16 months, respectively, compared with 34 and 15 months in the group receiving only NACT. In addition, the incidence of grade 3-4 adverse events is comparable between the two groups.

**Conclusion:**

This study supports the notion that the treatment regimen of NHIPEC combined with NACT can improve tumor response in advanced ovarian cancer compared with NACT alone.

## Introduction

High-grade serous ovarian cancer (HGSOC) is the most common and aggressive subtype of epithelial ovarian cancer. Despite advances in treatment, HGSOC remains a significant clinical challenge, with a 5-year survival rate of only about 20-60% due to late-stage diagnosis and high recurrence rates (1, 2). The standard treatment involves cytoreductive surgery combined with platinum-based chemotherapy, either as the initial therapy or after neoadjuvant chemotherapy (NACT) in cases of unresectable disease or high surgical risk (3). Interval debulking surgery (IDS) after NACT has shown better rates of complete tumor removal and lower surgical morbidity compared to primary debulking surgery (4–7). However, long-term survival outcomes are still limited, with many patients experiencing disease progression or recurrence (8), highlighting the urgent need for new therapeutic strategies.

Since HGSOC lesions are predominantly restricted to the peritoneal cavity, hyperthermic intraperitoneal chemotherapy (HIPEC), which delivers heated chemotherapy directly into the peritoneal cavity, has gained attention as a potential means to enhance drug penetration and cytotoxicity (9). The rationale for HIPEC is supported by both its thermal enhancement of chemotherapy efficacy and the pharmacokinetic advantage of high intraperitoneal drug concentrations (10–12). HIPEC was initially developed to treat patients with gastrointestinal malignancies accompanied by peritoneal metastases. A phase II clinical trial of gastric cancer conducted by Yang et al. first verified that this therapy could significantly enhance the clearance rate of peritoneal metastases and improve the complete resection rate of surgery (13, 14). This successful experience has spurred the exploration of HIPEC application in ovarian cancer. The OVHIPEC study carried out by the van Driel team (the first randomized controlled trial of HIPEC for ovarian cancer) confirmed that the combination of cisplatin HIPEC (100 mg/m^2^) after IDS could significantly extend the median progression-free survival (PFS: 14.2 vs. 10.7 months, P=0.003) and overall survival (OS: 45.7 vs. 33.9 months, P=0.02) of patients, and did not elevate the incidence of grade 3-4 ADEs (27% vs. 25%) (15). Similarly, a multicenter, retrospective cohort study reported prolonged OS with HIPEC in advanced epithelial ovarian cancer, reinforcing its therapeutic potential (16). More recent meta-analyses further support these findings, suggesting that HIPEC may be particularly beneficial in patients with optimal cytoreduction (17). Based on the results of this landmark study, HIPEC has been included as a recommended treatment for advanced ovarian cancer in international guidelines such as ESMO-ESGO and NCCN (18, 19).

Building on these advances, neoadjuvant HIPEC (NHIPEC), administered before NACT, has emerged as an innovative strategy to enhance tumor response and reduce peritoneal disease burden. Preliminary studies indicate that additional NHIPEC before NACT may improve pathological response rates (20, 21). Given the limitations of current therapies and the relatively small sample size of NHIPEC, an expanded evaluation of NHIPEC’s safety and survival benefits is warranted. Therefore, this study aimed to further assess the potential advantages of the combined regimen of NHIPEC and NACT, compared to NACT alone, in treating Chinese patients with HGSOC and to evaluate the safety.

## Methods

### Population

This study strictly adheres to the requirements of the Declaration of Helsinki and internationally accepted regulations. Although the Clinical Research Ethics Committee of the Cancer Hospital Affiliated to Zhengzhou University (Approval No: 2021-KY-0178) waives the need for informed consent (due to the retrospective nature of the analysis and no additional risks to patients), all patient data are anonymized. Patient identifiers such as names, ID card numbers, and hospitalization numbers are removed from the research data, and a coding system is used to replace personal information. We systematically reviewed and identified all patients who received NACT combined with IDS or NHIPEC+NACT combined with IDS at the Department of Gynecological Oncology of the Affiliated Cancer Hospital of Zhengzhou University from January 2016 to December 2018.

### Inclusion criteria

The inclusion criteria for this study are as follows: (1) patients diagnosed with high-grade serous epithelial ovarian cancer (including fallopian tube cancer and primary peritoneal cancer) and (2) disease stage conforming to the criteria of stage IIIC or IV set by the International Federation of Gynecology and Obstetrics (FIGO). Patients who do not meet the main treatment requirements or who have only received chemotherapy without completing the comprehensive treatment regimen are excluded.

### Treatment

For patients with a preliminary evaluation suggesting that achieving R0 resection via primary tumor cytoreductive surgery (PDS) would be challenging, we utilized the Suidan score or rapid intraoperative laparoscopic frozen section analysis in conjunction with resectability assessment based on the Fagotti scoring system. Patients with a Fagotti score < 8 undergo direct PDS, whereas those with a Suidan score > 3 or a Fagotti score ≥ 8 initially receive NACT. The therapeutic algorithm comprises up to three cycles of intravenous chemotherapy or a selective approach involving one cycle of NHIPEC, followed by an additional two cycles of intravenous NACT tailored to the patient’s clinical status. The drug dosage is strictly adjusted based on individualized factors. Paclitaxel is administered via intravenous infusion at a dose of 135-175 mg/m^2^ (175 mg/m^2^ for patients under 70 years old and 135 mg/m^2^ for those over 70 years old), with the infusion lasting 3 hours. This is followed by cisplatin, which is administered via HIPEC at a dose of 70-75 mg/m^2^. A continuous circulation mode is employed during perfusion, with a flow rate of 300-500 mL/min, precise intraperitoneal temperature maintenance at 43.0 ± 0.1°C, and a total perfusion duration of 60-90 minutes. All patients received mandatory intravenous hydration therapy, which was maintained for 24 h post-NHIPEC. The intravenous NACT protocol predominantly consists of platinum-based compounds combined with taxane derivatives. Adverse events (ADEs), predominantly manifesting during the first cycle of NACT, were graded and evaluated according to the most recent guidelines from the National Cancer Institute Common Terminology Criteria for Adverse Events. Patients demonstrating clinical responsiveness to NACT or stable disease status undergo IDS after completing the NACT. Next, all patients are mandated to receive a minimum of three cycles of adjuvant chemotherapy. In cases of disease progression detected during NACT, IDS is deemed contraindicated, and second-line chemotherapy regimens or palliative care interventions are recommended.

### Statistical analysis

We use the Chemotherapy Response Score (CRS) system to evaluate the pathological efficacy of NACT. The specific grading is as follows: CRS1, no or minimal tumor response; CRS2, detectable tumor regression; and CRS3, complete or near-complete remission with only a small number of residual tumor cells. The CRS was evaluated in a double-blind manner by two independent pathologists. In cases of disagreement between the two evaluators, a third senior pathologist conducted a review to determine the final result. We used standardized statistical methods to describe the demographic and clinical characteristics of patients and applied multivariate logistic regression analysis to identify independent predictors of CRS3. Progression-free survival (PFS) is defined as the period from the completion of the main treatment to disease progression, and overall survival (OS) is calculated from the completion of the main treatment until death. The Kaplan–Meier method was used to estimate PFS and OS, and the Cox proportional hazards model was used to calculate the hazard ratio to identify independent prognostic factors. All results were expressed with a 95% confidence interval (CI), and a P value<0.05 was considered statistically significant.

## Results

### Characteristics of the included cohort

A total of 123 patients received NACT and met the established inclusion criteria. Notably, 2 patients in the NHIPEC+NACT group and 1 patient in the NACT alone group experienced disease progression during neoadjuvant treatment. Thus, data from 120 patients were included in this study. **Table 1** summarizes the demographic information and clinical characteristics of the included cohort. The average age of the population is 54 years. 67 patients (55.83%) received the treatment regimen of NHIPEC+NACT. All patients in the NHIPEC+NACT group achieved the thermal uniformity standard after receiving NHIPEC, ensuring the effectiveness and safety of the treatment. Next, they received NACT within 3-4 weeks after NHIPEC, and there was no dose reduction during the entire treatment process, guaranteeing the integrity of the treatment plan and patient compliance. Specifically, 60 patients had a regular interval of 3 weeks (89.6%), and 7 patients had an interval extended to 4 weeks due to physical status (10.4%).

**Table 1:** Demographic and baseline disease characteristics of the study cohort.

Variable	NACT (n=53)	NHIPEC+NACT (n=67)	Overall (n=120)	P value
Age	51.54 ± 10.68	56.57 ± 8.16	54.39 ± 9.63	
BMI	22.42 ± 3.79	22.59 ± 3.53	22.52 ± 3.63	0.83
FIGO stage				<0.001
3	30 (25.00%)	58 (48.33%)	88 (73.33%)	
4	23 (19.17%)	9 (7.50%)	32 (26.67%)	
CRS				0.53
1	15 (12.50%)	15 (12.50%)	30 (25.00%)	
2	26 (21.67%)	31 (25.83%)	57 (47.50%)	
3	12 (10.00%)	21 (17.50%)	33 (27.50%)	
Pre-NACT CA125 (U/mL)	1537.47 ± 1431.73	1671.93 ± 1575.94	1612.54 ± 1509.20	0.35
Post-NACT CA125 (U/mL)	40.72 ± 41.94	265.46 ± 749.27	166.20 ± 569.82	<0.001
Pre-NACT HE4 (pmol/L)	657.85 ± 415.12	660.87 ± 440.78	659.53 ± 427.86	0.53
Post-NACT HE4 (pmol/L)	128.92 ± 219.07	184.53 ± 226.16	159.76 ± 223.81	0.26
Pre-NACT CRE (μmol/L)	51.64 ± 8.92	52.67 ± 8.79	52.22 ± 8.83	0.62
Post-NACT CRE (μmol/L)	50.79 ± 11.48	53.55 ± 20.51	52.32 ± 17.08	0.44
Pre-NACT Hb (g/L)	103.53 ± 12.92	103.06 ± 13.58	103.27 ± 13.24	0.78
Post-NACT Hb (g/L)	100.96 ± 14.93	100.28 ± 15.30	100.58 ± 15.08	0.67
R0				<0.001
Yes	37 (30.83%)	67 (55.83%)	104 (86.67%)	
No	16 (13.33%)	0 (0%)	16 (13.33%)	

NACT, neoadjuvant chemotherapy; NHIPEC, neoadjuvant hyperthermic intraperitoneal chemotherapy; BMI, body mass index; FIGO, international federation of gynecology and obstetrics; CRS, cytoreductive surgery, CA125, cancer antigen 125; HE4, human epididymis protein 4; CRE, creatinine; Hb, hemoglobin; R0, resection with no residual tumor.

### Impact of NHIPEC on CRS and prognosis

**Figure 1:**
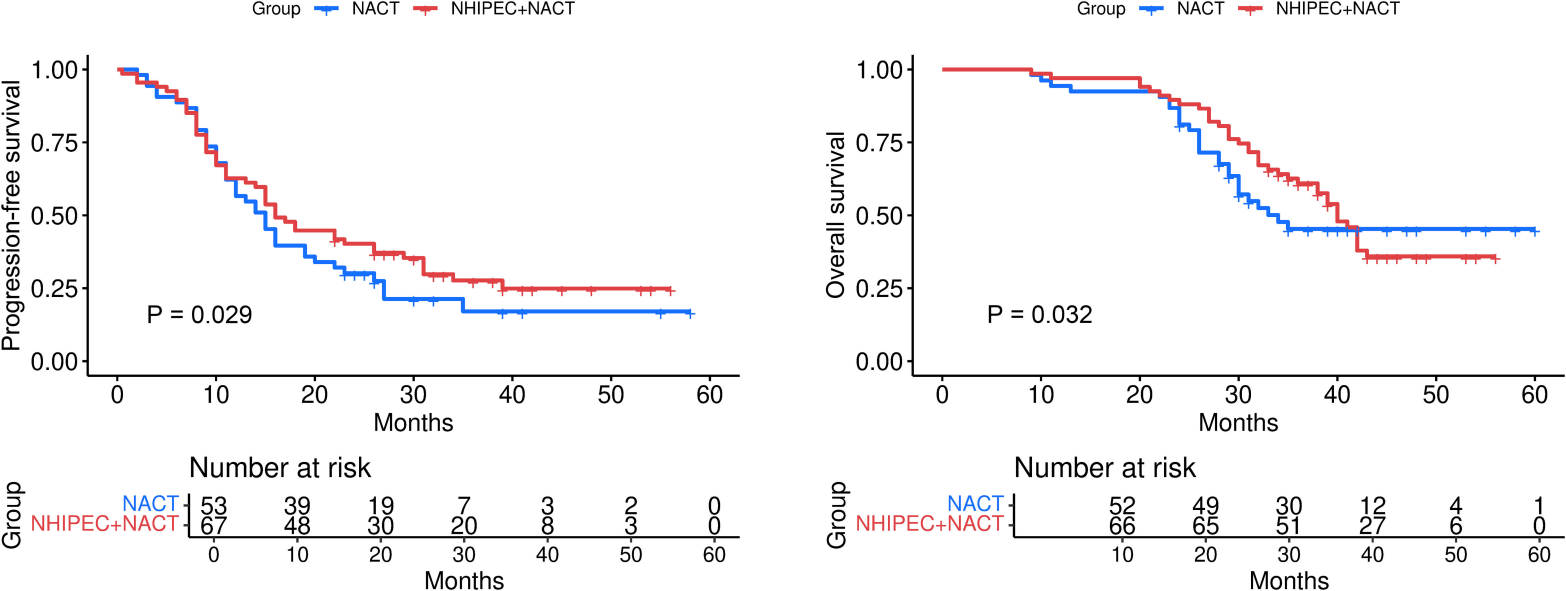
Kaplan-Meier curves comparing progression-free survival (PFS) and overall survival (OS) between patients receiving neoadjuvant chemotherapy (NACT) alone and those receiving neoadjuvant hyperthermic intraperitoneal chemotherapy (NHIPEC) combined with NACT (NHIPEC+NACT). The median PFS was 15 months in the NACT group vs. 16 months in the NHIPEC+NACT group (Cox proportional hazards model, P=0.029) (Left panel). The median OS was 34 months in the NACT group vs. 40 months in the NHIPEC+NACT group (Cox proportional hazards model, P=0.032) (Right panel). The number at risk is provided below the curves to indicate the number of patients remaining in follow-up at each time point.

After NACT, 33 patients achieved CRS3. The proportion of patients achieving CRS3 is higher in the NHIPEC+NACT group than in the NACT group (31% vs. 23%, respectively) (**Table 1**). Given the significant role of the CRS in the prognostic assessment of patients with HGSOC, we explored the potential predictors of CRS3 using ordered logistic regression analysis. The results of the analysis are presented in **Table 2**. We identified NHIPEC (HR=2.31, 95%CI: 1.74-2.88, P=0.002) as the key factor independently associated with CRS3. Multivariate Cox regression analysis showed that NHIPEC, CRS, and post-NACT CA125 levels are significantly negatively correlated with the risk of disease progression and death in patients (**Tables 3**, **4**). NHIPEC is an independent favorable prognostic factor for OS (P=0.032) and PFS (P=0.029) (**Tables 3**, **4**). The median OS and PFS of patients receiving the combined treatment regimen were extended to 40 and 16 months, respectively, compared with 34 and 15 months in the group receiving only NACT (**Figure 1**). These data indicate that adding NHIPEC to NACT can improve patient outcomes. However, we observed a cross-trend in the OS curves, indicating that the NHIPEC+NACT group exhibited early benefits, while the NACT alone group may have shown improved performance in later stages. This observation implies a more pronounced early tumor response to NHIPEC, which is consistent with the higher percentage of CRS3 in the combination group (17.5% compared to 10.0%). The reasons for the relatively lower long-term survival rate in the combined group remain unclear. This may be associated with differences in postoperative treatment management or gene mutation status of patients. The contribution of these factors to the long-term prognosis of NHIPEC requires further investigation in expanded samples.

**Table 2:** The ordered logistic multivariate regression analysis of CRS.

Variate	P value	Hazard ratio
FIGO stage	0.281	0.660 (0.310-1.400)
Age	0.106	1.030 (0.990-1.070)
BMI (kg/m2)	0.549	0.970 (0.890-1.060)
Pre-NACT CA125 (U/mL)	0.728	1.000 (1.000-1.000)
Post-NACT CA125 (U/mL)	0.737	1.000 (1.000-1.000)
Pre-NACT HE4 (pmol/L)	0.160	1.000 (1.000-1.000)
Post-NACT HE4 (pmol/L)	0.433	1.000 (1.000-1.000)
Pre-NACT CRE (μmol/L)	0.095	1.030 (0.990-1.080)
Post-NACT CRE (μmol/L)	0.137	1.020 (0.990-1.040)
Pre-NACT Hb (g/L)	0.624	1.010 (0.980-1.030)
Post-NACT Hb (g/L)	0.850	1.000 (0.980-1.020)
NHIPEC	0.002	2.310 (1.740-2.880)

NACT, neoadjuvant chemotherapy; NHIPEC, neoadjuvant hyperthermic intraperitoneal chemotherapy; BMI, body mass index; FIGO, international federation of gynecology and obstetrics; CRS, cytoreductive surgery, CA125, cancer antigen 125; HE4, human epididymis protein 4; CRE, creatinine; Hb, hemoglobin; R0, resection with no residual tumor.

**Table 3:** The Cox multivariate regression analysis of PFS.

Variate	P value	Hazard ratio
FIGO stage	0.542	1.197 (0.671-2.133)
Age	0.958	1.001 (0.976-1.026)
BMI (kg/m^2^)	0.179	1.043 (0.981-1.109)
Pre-NACT CA125 (U/mL)	0.059	1.000 (1.000-1.000)
Post-NACT CA125 (U/mL)	0.004	1.001 (1.000-1.002)
Pre-NACT HE4 (pmol/L)	0.883	1.000 (0.999-1.001)
Post-NACT HE4 (pmol/L)	0.123	1.001 (1.000-1.002)
Pre-NACT CRE (μmol/L)	0.510	1.010 (0.981-1.040)
Post-NACT CRE (μmol/L)	0.073	0.983 (0.964-1.002)
Pre-NACT Hb (g/L)	0.792	1.003 (0.980-1.027)
Post-NACT Hb (g/L)	0.512	0.994 (0.974-1.013)
NHIPEC	0.029	0.455 (0.153-0.756)
CRS	0.007	0.541 (0.286-0.796)
R0	0.221	0.645 (0.320-1.302)

NACT, neoadjuvant chemotherapy; NHIPEC, neoadjuvant hyperthermic intraperitoneal chemotherapy; BMI, body mass index; FIGO, international federation of gynecology and obstetrics; CRS, cytoreductive surgery, CA125, cancer antigen 125; HE4, human epididymis protein 4; CRE, creatinine; Hb, hemoglobin; R0, resection with no residual tumor.

**Table 4:** The Cox multivariate regression analysis of OS.

Variate	P value	Hazard ratio
FIGO stage	0.048	3.476 (1.009-11.975)
Age	0.381	1.021 (0.975-1.070)
BMI (kg/m2)	0.081	0.886 (0.774-1.015)
Pre-NACT CA125 (U/mL)	0.121	1.000 (1.000-1.001)
Post-NACT CA125 (U/mL)	0.004	1.001 (1.000-1.002)
Pre-NACT HE4 (pmol/L)	0.774	1.000 (0.999-1.001)
Post-NACT HE4 (pmol/L)	0.198	0.998 (0.995-1.001)
Pre-NACT CRE (μmol/L)	0.073	0.944 (0.886-1.005)
Post-NACT CRE (μmol/L)	0.450	0.988 (0.957-1.020)
Pre-NACT Hb (g/L)	0.560	1.013 (0.971-1.057)
Post-NACT Hb (g/L)	0.352	0.981 (0.941-1.022)
NHIPEC	0.032	0.591 (0.346-0.835)
CRS	0.019	0.568 (0.316-0.821)
R0	0.330	0.463 (0.099-2.174)

NACT, neoadjuvant chemotherapy; NHIPEC, neoadjuvant hyperthermic intraperitoneal chemotherapy; BMI, body mass index; FIGO, international federation of gynecology and obstetrics; CRS, cytoreductive surgery, CA125, cancer antigen 125; HE4, human epididymis protein 4; CRE, creatinine; Hb, hemoglobin; R0, resection with no residual tumor.

### Impact of NHIPEC on the incidence of perioperative ADEs

**Figure 2:**
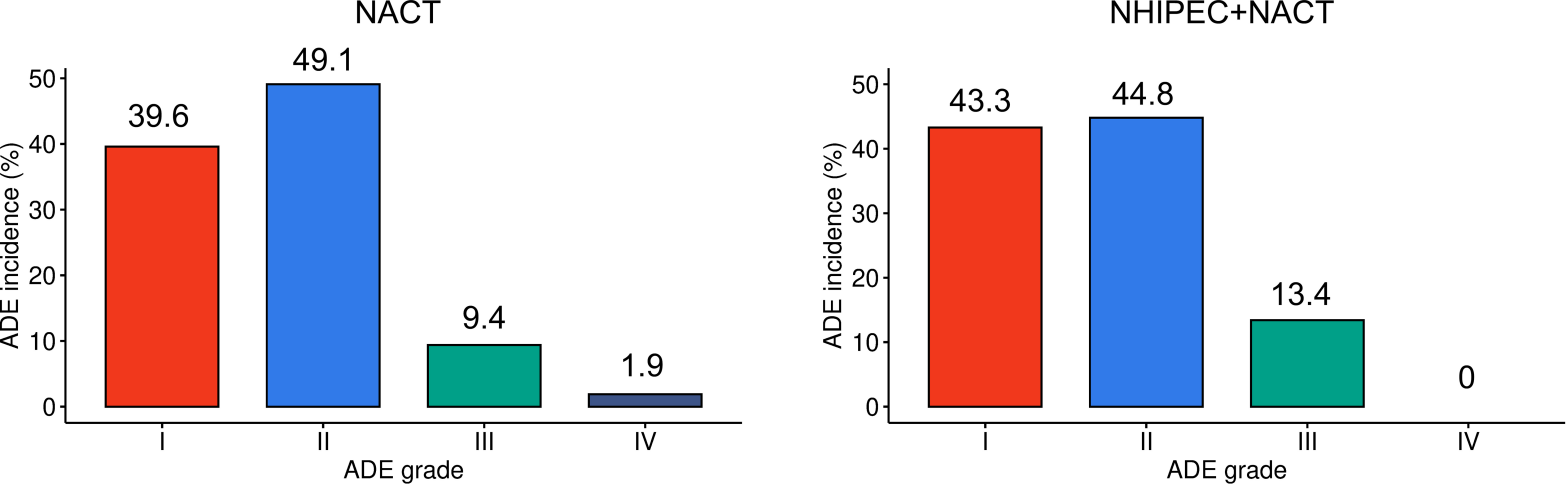
Bar chart comparing the incidence of perioperative adverse events (ADEs) between the neoadjuvant chemotherapy (NACT) and neoadjuvant hyperthermic intraperitoneal chemotherapy (NHIPEC)+NACT groups. ADEs are graded according to the National Cancer Institute Common Terminology Criteria for Adverse Events. The overall incidence of grade III-IV ADEs was 11.3% in the NACT group and 13.4% in the NHIPEC+NACT group. The comparison of ADE incidences was performed using Fisher’s exact test, and there is no statistical significance.

**Table 5** summarizes the ADEs observed within the first 3 weeks of the first NACT treatment cycle. Among patients who received NHIPEC+NACT, no case of grade 4 severe ADEs was reported, demonstrating the satisfactory performance of this combination therapy in terms of safety. Further comparison revealed that among patients receiving NACT alone, the incidence of grade III-IV ADEs reaches 11.3% (**Figure 2**). In contrast, among patients in the NHIPEC+NACT group, the incidence of ADEs of this grade is 13.4% (**Figure 2**). The difference is not statistically significant. These data indicate that NHIPEC does not significantly affect the life quality of patients.

**Table 5:** ADEs within three weeks of the first cycle of NACT.

ADEs	NACT (n=53)				NHIPEC+NACT (n=67)			
	I	II	III	IV	I	II	III	IV
Thrombocytopenia	1	2	0	0	2	1	0	0
Leukopenia	4	6	4	1	5	3	5	0
Diarrhea	2	2	0	0	1	2	0	0
Abdominal pain	3	3	0	0	8	7	0	0
Vomiting	5	4	0	0	4	6	0	0
Fever	1	1	0	0	1	2	0	0
Infection	0	0	1	0	0	0	3	0
Thrombosis	0	3	0	0	0	4	0	0
Creatinine	2	0	0	0	3	0	0	0
Transaminase	1	2	0	0	2	1	1	0
Electrolyte disturbance	2	3	0	0	3	4	0	0

NACT, neoadjuvant chemotherapy; NHIPEC, neoadjuvant hyperthermic intraperitoneal chemotherapy; ADE, adverse event.

## Discussion

Our study focuses on the NHIPEC with the intention to treat preoperative bulky tumors, where vasculature remains intact, allowing high concentrations of intraperitoneal drugs to act directly on the primary tumor, whereas postoperative HIPEC is more focused on eliminating residual micrometastasis. We retrospectively evaluated the effect of HIPEC applied before NACT and found that it increased the rate of CRS3 after cytoreductive surgery compared with single NACT. Multivariate regression analysis indicated that NHIPEC is an independent favorable prognostic factor for CRS3 (P=0.002), OS (P=0.032), and PFS (P=0.029), supporting its application in the neoadjuvant setting. Additionally, there was no statistically significant difference in the incidence of perioperative ADEs (including grade III-IV) between the two patient groups.

Recently, data from a multicenter retrospective cohort study on NHIPEC have shown that NHIPEC has potential in improving chemotherapy response (P=0.033) and PFS (22 vs. 16 months, P<0.001) (21), which is similar to the results of our study. When we were preparing this manuscript, Sun et al. reported that NHIPEC combined with NACT can significantly increase the rate of achieving CRS3 (42.4% vs 18.9%, P=0.03) in HGSOC (22). They also reported that NHIPEC is well-tolerated, with no grade 4 ADE, and the incidence of grade 3 ADEs is 14% (mainly neutropenia and vomiting). The main ADEs in our study are similar to theirs, but the incidence of grade 3 ADEs is lower. Regarding the cisplatin dose, although hyperthermia may increase nephrotoxicity (a 15–20% decrease in creatinine clearance) by reducing renal tubular excretion (23), there is currently no international consensus: the NCCN guidelines recommend 100 mg/m^2^ (24), while the OVHIPEC-2 trial adopts a 75 mg/m^2^ regimen (25). In the data of this study, we generally used a dose of 75 mg/m^2^. For individual patients, a dose of 70 mg/m^2^ was administered based on their physical conditions. In the NHIPEC group, no significant changes in serum creatinine levels were observed.

Hyperthermia can strengthen the antitumor effect through multiple mechanisms. First, the thermokinetic effect can increase the permeability of the cell membrane, raising the intracellular concentration of cisplatin by 3-5 times (26). Second, heat stress can induce apoptosis of tumor cells (with increased expression of Caspase-3) and upregulate heat shock protein (HSP70) to activate the killing effect of NK cells (27, 28). Through single-cell RNA sequencing analysis, Sun et al. found that tumor cells with epithelial-mesenchymal transition and MMP-11+ cancer-associated fibroblasts are more sensitive to NHIPEC, and NHIPEC can improve the immunosuppressive tumor microenvironment, enhancing the anti-tumor effect of PD-L1 antibodies in HGSOC. Additionally, hyperthermia can inhibit VEGF-mediated angiogenesis (resulting in a 58% reduction in microvessel density) and directly kill tumor cells through protein denaturation (29). From the pharmacokinetic perspective, with intraperitoneal administration, the drug can act directly on the local peritoneal cavity and form a high-concentration accumulation on the peritoneal surface (30), while the amount of drug entering the systemic circulation is relatively small. In contrast, intravenous administration requires distribution through the systemic blood circulation, and the drug reaching the peritoneal surface is significantly reduced due to peripheral metabolism, with a higher systemic exposure (31). This unique feature of ‘high local concentration-low systemic toxicity’ renders HIPEC an ideal regional treatment approach.

A treatment temperature range of 41-43°C can selectively induce apoptosis of tumor cells via the thermal effect while effectively safeguarding the physiological functions of normal tissues. This differential thermal sensitivity offers a molecular-level elucidation of the safety of HIPEC (32). The third-generation hyperthermia perfusion systems can achieve precise temperature control within ±0.1°C, ensuring that the temperature of the perfusion fluid is stably maintained within the treatment range (33, 34). In the clinical observation of NHIPEC conducted by our team and others, through standardized operation procedures and a real-time monitoring system, there was no significant difference in the incidence of perioperative ADEs between the treatment and control groups (20). This outcome further validates the safety of NHIPEC under standardized operating conditions. Considering the existing clinical evidence and the current state of technical development, it can be affirmed that, on the premise of strictly adhering to the operation specifications and ensuring the precision of temperature control and the monitoring of drug concentration, NHIPEC, as a crucial component of the comprehensive treatment of advanced tumors, can enhance the tumor debulking effect and has its safety fully authenticated, thereby laying a solid foundation for its application in the neoadjuvant settings. However, as a retrospective cohort study, our study has inherent selection bias (e.g., fewer patients with FIGO stage IV in the NHIPEC group) and a limited single-center sample size, which may affect the extrapolation of the results. Therefore, the results of our study need to be validated by multicenter, large-sample randomized controlled trials. Currently, they can only serve as a reference for clinical practice rather than a recommended standard.

## Conclusion

The findings of this study demonstrated that, compared with the NACT monotherapy regimen, the combination of NHIPEC and NACT can improve the rate of achieving optimal tumor debulking (CRS3) in patients with HGSOC. Regarding survival outcomes, the median OS and PFS of patients receiving the combined treatment regimen were extended to 40 and 16 months, respectively, compared with 34 and 15 months in the group receiving only NACT. These results suggest that NHIPEC combined with NACT may confer potential clinical benefits to HGSOC patients by enhancing tumor debulking efficacy. Nevertheless, survival outcomes require further evaluation through randomized controlled trials with larger sample sizes.

## Data Availability

The raw data supporting the conclusions of this article will be made available by the authors, without undue reservation.
